# 

**DOI:** 10.1192/bjb.2022.86

**Published:** 2023-12

**Authors:** Linda Gask

**Affiliations:** is a writer and Professor Emerita of Primary Care Psychiatry in the Centre for Primary Care, University of Manchester, Manchester, UK. Email: linda.gask@manchester.ac.uk

**Figure:**
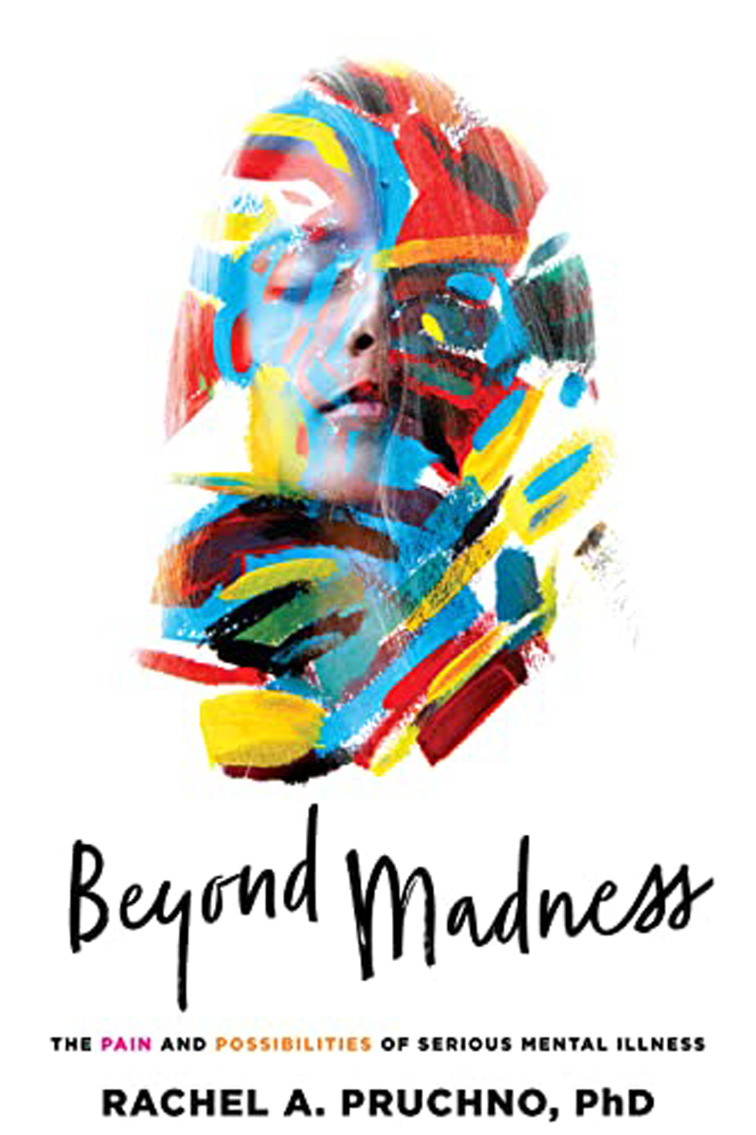

In *Beyond Madness* Rachel Pruchno, a developmental psychologist, writes what she has learned about severe mental illness: its impact on people and their families and what does, and doesn't, help with recovery. She tells stories of real people, including that of 17-year-old Joe, diagnosed with schizophrenia, who has figured out that Satan has disguised himself as his own Grandma and is planning to kill him – because Joe is now God; social worker Sharon, recently diagnosed with depression and considering suicide; and Michelle, whose sudden powerful bursts of energy and ability to manage with no sleep eventually are diagnosed as bipolar disorder. She also tells us how her own adoptive daughter Sophie's problems started in childhood. How she acquired multiple diagnoses, beginning with ADHD and then several ‘comorbidities’ too. How she suffered polypharmacy, repeated hospital admissions and eventually became estranged from her family as an adult on finding it hard to continue taking medication for the now diagnosed bipolar disorder.

All these tales, and more, take the reader through the awful reality of the dysfunctional American mental healthcare ‘system’. As Pruchno sees it, there is not only a failure to understand the nature and impact of severe mental illness, with confusion between models of understanding and providing care, but also a frightening disconnect between professionals and organisations, who don't get rewarded by insurance companies to communicate with one other. Half of those who suffer receive no help at all and many try to survive on the streets or are incarcerated in prison. Senseless, when we know a great deal about what could be done to help them.

Woven between is what Pruchno has come to understand about severe mental illness, which she unapologetically describes as a ‘brain disorder’. She interviews Dr E. Fuller Torrey, the venerable psychiatric researcher, who tells her about his own family experience of schizophrenia. Multiple ways are listed, and discussed, for families, friends and communities to help people overcome the obstacles to getting care, although these largely describe what is available in the USA (apart from a brief diversion to Trieste). The author also has a rosier view than most of us would recognise of how crisis services function in the UK. There is a touching section on how and what to tell others about family illness, based not only on Pruchno's more recent experiences with her daughter but also coincidentally on growing up herself with a mother with bipolar disorder.

Finally, she tells how, on a visit to Israel in 2012, a scribe in a synagogue overlooking the Dead Sea gave her a note with a message to give to her daughter when she finally came home.

Sadly, in 2021, she still had that note.

